# Experimental verification of the standard model of particle physics

**DOI:** 10.2183/pjab.97.013

**Published:** 2021-05-11

**Authors:** Tomio KOBAYASHI

**Affiliations:** *1 International Center for Elementary Particle Physics (ICEPP), The University of Tokyo, Hongo 7-3-1, Bunkyo-ku, Tokyo 113-0033, Japan.

**Keywords:** standard model, gluon, Higgs boson, supersymmetry

## Abstract

The history concerning an experimental verification of the standard model of particle physics is reviewed with special emphasis on results from experiments using the highest-energy particle colliders, namely, PETRA, LEP and LHC. This article covers physics subjects from discovering the gluon and precise measurements at LEP, to discovering the Higgs boson. It also covers some searches for physics beyond the standard model, particularly supersymmetry, as well as recent developments of some particle detectors that were used in those experiments.

## 1 Introduction

All theoretical ingredients for the standard model of particle physics were available by 1974, and were ready to be scrutinized by experiments. During 40 years thereafter, a number of discoveries and fine measurements were made, and the standard model became established.

The standard model of particle physics is based on theories that describe the elementary particles and their interactions, namely the electro-magnetic, weak and strong forces.

The present particle contents of the standard model are given in Table 1 (fermions) and in Table 2 (bosons). The particles in the hatched areas were not found before 1974. Fermions (spin 1/2) were supposed to be the basic constituents of all material in the universe. Vector bosons (spin 1) are carriers of the basic forces of the standard model, and a scaler boson (spin 0) is responsible for the mass of all particles in the standard model.

The fundamental theory of the standard model is the gauge theory. The electro-magnetic interaction is described by the U(1) gauge theory: quantum electrodynamics (QED). The electro-magnetic force and the weak force are combined into the electro-weak (EW) force in the SU(2) × U(1) gauge theory, giving a massless photon (γ) and massive weak bosons (*W*^±^, *Z*^0^). The Higgs mechanism was introduced in the EW theory, so as to give masses to the standard-model particles, and as a consequence a spinless neutral Higgs boson (*H*) should exist. The strong force between quarks, and hence between hadrons, is described by SU(3) gauge theory with 3 color charges. This theory, called quantum chromodynamics (QCD), predicts the existence of gluons as the carrier of the strong force. But none of the bosons, except for the photon, were found before 1974.

The fermionic part of the standard model consists of quarks, which have all 3 interactions (*i.e.*, electro-magnetic, weak and strong forces), and leptons, which have 2 interactions other than the strong interaction. Quarks, as well as leptons, appear as a pair (like u and d, or ν_*e*_ and e) to form an SU(2) doublet of the weak interaction. Fermions in the 1-st generation are supposed to form almost all of the materials in the universe, since stable atoms or nuclei are made of them.

Until 1974, all of the hadrons, including the strange particles, were explained by the quark model with 3 quarks (u, d and s). While the lepton doublet of the 2-nd generation (ν_μ_, μ) was already found, the counterpart of the s-quark was missing. The GIM mechanism^1)^ was proposed to cure a strangeness-changing neutral-current problem, which predicted the existence of a 4-th quark (charm, c).

In November 1974, the *J*/ψ particle was discovered at Brookhaven National Laboratory (BNL) and at Stanford Linear Accelerator Center (SLAC). Soon afterwards the *J*/ψ was confirmed to be a ground-state charmonium, which is a meson composed of c and $\bar{\text{c}}$ (anti-particle of c quark). The other charmonium states as well as the open charm states (both charmed mesons and charmed baryons) were discovered one after another, so that the apparent flaw of the standard model framework had been removed. Hence, the discovery of *J*/ψ was called the “November Revolution”, and it was the beginning of an experimental verification of the standard model of particle physics.

In 1975 the τ lepton was discovered at SLAC, and in 1977 the Υ particle (the ground-state bottomonium b$\bar{\text{b}}$) was discovered at Fermi National Accelerator Laboratory (Fermilab), which strongly indicated the existence of 3-rd generation fermions.

The International Center for Elementary Particle Physics (ICEPP) was established in 1974 at the University of Tokyo in order to participate in the DASP experiment using the DORIS accelerator at Deutsches Elektronen Synchrotron (DESY). DORIS is a positron-electron (*e*^+^*e*^−^) collider of which the maximum center-of-mass (c.m.) energy ($\sqrt{s}$) was 11.2 GeV. The ICEPP team made an essential contribution to the discovery of the *P*_*c*_ particle, one of the charmonium states.

DESY then decided to build the world highest energy *e*^+^*e*^−^ collider, PETRA ($\sqrt{s}=46.8$ GeV max.), of which one of the main objectives was to find the top quark (t), a counterpart of the b quark in the 3-rd generation quark doublet. PETRA completed its construction and started operation in 1978. The ICEPP team formed the international group, the JADE collaboration, together with German and UK teams to participate in experiments at PETRA.

The author joined the ICEPP team as a research staff member in 1977, and participated in the JADE experiment. The activities of ICEPP continued from experiments at DESY to two experiments at the European Organization for Nuclear Research (CERN): OPAL experiment using LEP *e*^+^*e*^−^ collider from 1980, and ATLAS experiment using the LHC proton-proton collider from 1992. The author was involved in all 3 experiments (JADE, OPAL and ATLAS) until 2015.

This article describes the important findings made by the experiments at PETRA, LEP and LHC, as well as those from other experiments, in the light of an experimental verification of the standard model of particle physics.

The standard model was known to have its own limitation, namely: i) it does not have the gravity interaction, ii) it has a charge quantization problem, iii) too many parameters, iv) a hierarchy problem, v) a generation problem, vi) no solutions to dark matter, dark energy, and baryon asymmetry in the universe, and so on. These necessitate a new theory which includes the standard model.

There are a number of theories which can solve some of these problems. Some of the candidates are: theories with supersymmetry, quark/lepton substructures, technicolor models, quantum gravity theories, *etc.* During the course of experimentally verifying the standard model of particle physics, especially the supersymmetry theories and the technicolor theories, these came up as the main candidate theories just beyond the standard model. In this article some of the searches for new physics beyond the standard model are also presented, with emphasis on these two types of theories. The details of these theories are briefly described in the relevant sections of this article.

## 2 PETRA period (1978–)

The PETRA (Positronen-Electronen Tandem Ring Anlage) *e*^+^*e*^−^ collider was constructed at DESY, and started operation in 1978. By the end of 1979, it provided *e*^+^*e*^−^ collisions between $\sqrt{s}=12$ GeV and 31.6 GeV, which gave rise to many important results. PETRA continued working until 1986, and its attained highest collision energy was 46.8 GeV.

**Figure 1.**
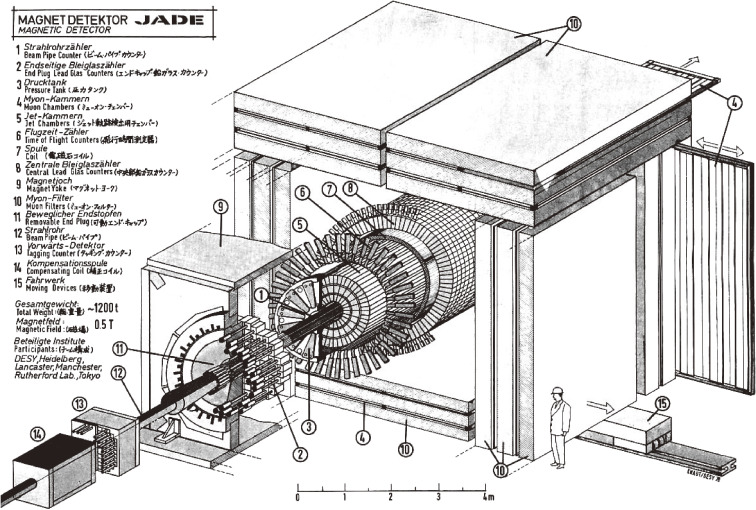
Sectional view of the JADE detector.

There were 4 experiments at PETRA: JADE, Mark J, TASSO and PLUTO (which was replaced by CELLO from 1980). A sectional view of the JADE detector is shown in Fig. 1. The ICEPP team was involved in the JADE experiment, and contributed to the construction and operation of the electro-magnetic shower calorimeter, which consisted of 2520 lead glass counters in the barrel part, and 192 lead glass counters on the two end caps of the detector.

### 2.1 Top quark search.

One of the main objectives of the PETRA experiments was to find the top quark (t). Many theorists predicted the t quark mass to be at around 13.5 GeV, mainly from the fact that the masses of s, c, b quarks are ∼0.5 GeV, ∼1.5 GeV, ∼4.5 GeV, respectively. The first results concerning the t quark search using the data taken until 1979 were given by MARK J,^2)^ PLUTO,^3)^ JADE,^4,5)^ and TASSO.^6)^

New flavor quark pair production in *e*^+^*e*^−^ collisions should enhance the multihadronic event rate, so that the search was made through measurements of the *R* value:

$$R=\frac{\sigma (e^{+}e^{-}\to \text{hadrons})}{\sigma (e^{+}e^{-}\to \mu^{+}\mu^{-})}.$$ [1]

It is equal to $3\sum_{i}\;q_{i}^{2}$ in the simple quark model, where *q*_*i*_ is the charge (in the unit of *e*) of the quark *i*, and the factor 3 comes from the number of quark colors. The results were consistent with the production of the known 5 quarks (u, d, s, c, b), and there was no sign of any excess concerning the R values.

All 4 experiments made another more sensitive search for the top quark, by looking into the event shape of the multihadronic events. Just above the production threshold of a new heavy quark pair, its event shape was supposed to be spherical, while the lighter quark pair productions would give 2-jet-like event shapes.

There were a number of event shape variables used in the analyses, such as the thrust (*T*), sphericity (*S*), spherocity (*S*′), *etc.*, which were defined as follows:

$$T=\max\left[\frac{\sum |p_{i\|}|}{\sum p_{i}}\right],$$ [2]

$$S=\min\left[\frac{\sum p_{i⊥}^{2}}{\sum p_{i}^{2}}\right],$$ [3]

$$S^{\prime }={\left(\frac{4}{\pi }\right)}^{2}\min\left[\frac{\sum p_{i⊥}}{\sum p_{i}}\right].$$ [4]

Here, *p*_*i*__∥_ and *p*_*i*__⊥_ are the longitudinal and transverse momenta of the hadron (*i*) relative to the jet axis, which is determined by varying the direction of this axis for each event.

The sphericity value can also be obtained by diagonalizing the sphericity tensor,

$$T_{\alpha \beta }=\frac{\sum_{i}p_{i\alpha }p_{i\beta }}{\sum_{i}p_{i}^{2}},$$ [5]

where *p*_*i*__α_ is the α-component (α = *x*, *y*, *z*) of the momentum of the *i*-th particle. From the resulting eigenvalues, *Q*_1_, *Q*_2_, *Q*_3_ (*Q*_1_ < *Q*_2_ < *Q*_3_, *Q*_1_ + *Q*_2_ + *Q*_3_ = 1), the sphericity value is calculated accordingly to

$$S=\frac{3}{2}(Q_{1}+Q_{2}),$$ [6]

and the sphericity axis is given by the principal axis of the momentum ellipsoid corresponding to the eigenvalue *Q*_3_.

**Figure 2.**
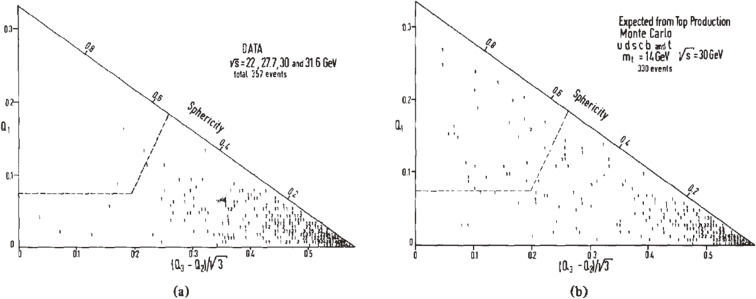
Distribution of the eigenvalues *Q*_1_, *Q*_2_, *Q*_3_ of the sphericity tensor. *Q*_1_ is plotted versus (*Q*_3_ − *Q*_2_)/$\sqrt{3}$. (a) Data from all energies combined and (b) model prediction at $\sqrt{s}=30$ GeV including top quark (*m*_*t*_ = 14 GeV) production. The dashed lines show the cuts *Q*_1_ > 0.075 and sphericity *S* > 0.55.

Figure 2(a) is a Dalitz plot (named the “Q-plot”) used by the JADE experiment,^5)^ where the perpendicular sides of the triangle are *Q*_1_ and (*Q*_3_ − *Q*_2_)/$\sqrt{3}$, so that the hypotenuse is the sphericity, *S*. Each event from the various c.m. energies ($\sqrt{s}$) is represented by a point in the plot. While the spherical events expected from the top quark production would give a wide distribution in the plot, no significant accumulation of the events was observed in the upper part of the triangle.

As a comparison, the results of a model calculation are shown in Fig. 2(b). This model is based on the quark-antiquark pair, $q\bar{q}$ (*q* = *u*, *d*, *s*, *c*, *b*, *t*), productions, assuming that the top quark decays through the chain *t* → *b* → *c* → *s*, and on the cascade mechanism from the quark fragmentation into hadrons. The top quark mass (*m*_*t*_) was fixed to 14 GeV, and the c.m. energy to 30 GeV in the Monte-Carlo simulation. The normalization for the number of events was based on the accumulated luminosity. In order to draw quantitative results, the observed and expected events in the regions *S* > 0.55 and *Q*_1_ > 0.075 were compared. A Monte-Carlo simulation without top quark production yields no events in this region.

In conclusion, no evidence was observed concerning the production of a top quark with *m*_*t*_ between 11 and 14 GeV. Similar results were also obtained by Mark J,^2)^ PLUTO^3)^ and TASSO^6)^ using various event shape variables.

**Figure 3.**
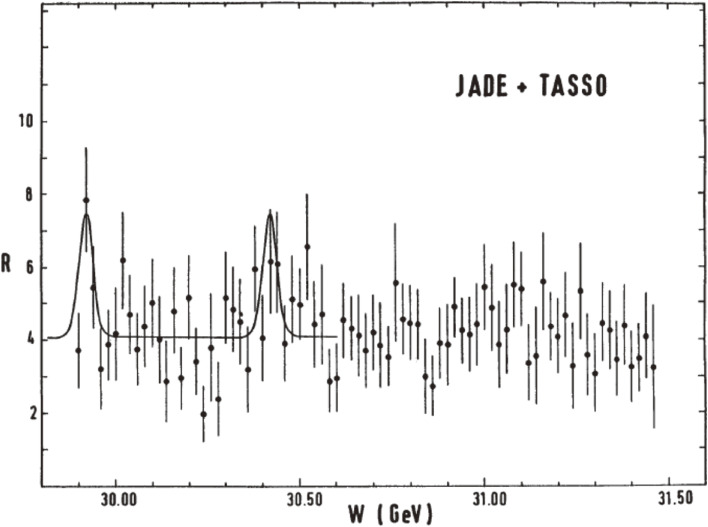
Values of *R* = σ(*e*^+^*e*^−^ → hadrons)/σ(*e*^+^*e*^−^ → μ^+^μ^−^) as a function of c.m. energy W. The results from JADE and TASSO data are summed.

Similar to the existence of *J*/ψ and Υ for c and b quarks, respectively, the models of heavy quarkonia predict the $t\bar{t}$ bound states to lie below the threshold for the $t\bar{t}$ continuum. The experiments at PETRA made fine energy scans to search for the narrow states at around the 30 GeV c.m. energy region, and at later stages up to the highest energies at PETRA. Figure 3 shows the energy scan results made by JADE and TASSO at around the 30 GeV energy region, where the *R* values (Eq. [1]) are plotted as a function of the c.m. energies.^7)^ No significant structure was seen.

PETRA increased the energy up to $\sqrt{s}=46.8$ GeV, and searched for the $t\bar{t}$ narrow resonance, but the results were negative.^8–10)^ The top-quark search was then taken over by AMY, TOPAZ and VENUS experiments using the *e*^+^*e*^−^ collider TRISTAN at the National Laboratory for High Energy Physics in Japan (KEK), of which the maximum attained energy was $\sqrt{s}=64$ GeV. The obtained result was excluding the top quark with a mass below ∼30 GeV.^11–13)^

### 2.2 Discovery of the gluon.

The most important result from the PETRA experiments was discovering the gluon, the carrier of the strong force.

Quark-parton models predicted the two-jet structure in multihadron production in the *e*^+^*e*^−^ annihilation reaction at higher energies, which was expected from the process *e*^+^*e*^−^ → $q\bar{q}$, with a subsequent fragmentation of the quarks in to hadrons. This phenomenon had been clearly observed by experiments at the SPEAR *e*^+^*e*^−^ storage ring ($\sqrt{s}=3.0\text{--}7.4$ GeV) of SLAC,^14)^ and by PLUTO at DORIS ($\sqrt{s}=3.1\text{--}9.5$ GeV) of DESY,^15)^ using the sphericity distributions.

**Figure 4.**
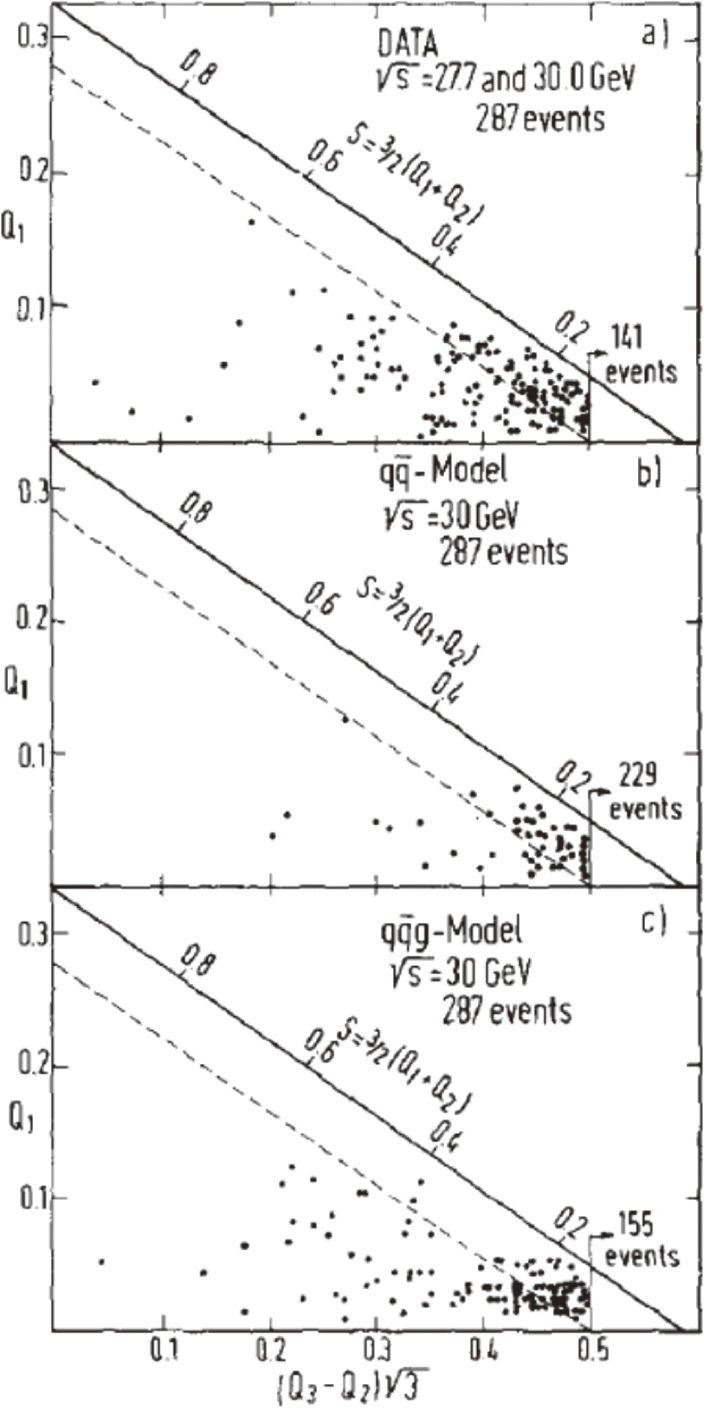
“Q-plot” (described in the text) for (a) data at $\sqrt{s}=27.7$ and 30.0 GeV, (b) $q\bar{q}$ model prediction, (c) $q\bar{q}g$ model prediction. The planarity (*Q*_2_ − *Q*_1_) axis is orthogonal to the sphericity axis. The dotted line indicates planarity = 0.07.

The QCD theory of strong interactions predicts a specific type of event structure deviating from the two-jet structure due to the gluon bremsstrahlung process, *e*^+^*e*^−^ → $q\bar{q}g$. This implicates that the data should contain events with planar or three-jet configurations. JADE looked into the Q-plot.^16)^ Figure 4(a) shows the data at $\sqrt{s}=27.7$ and 30.0 GeV, which can be compared with the $q\bar{q}$ model prediction, Fig. 4(b), and the $q\bar{q}g$ model prediction, Fig. 4(c). The transverse momentum (*q*_⊥_) of secondary quarks in the hadron-jet cascade is described by $d\sigma /d^{2}q_{⊥}∼\exp-q_{⊥}^{2}/2\sigma_{q}^{2}$, where σ_*q*_ was set to the standard value of 250 MeV. A significant deviation from the two-jet model is apparent.

**Figure 5.**
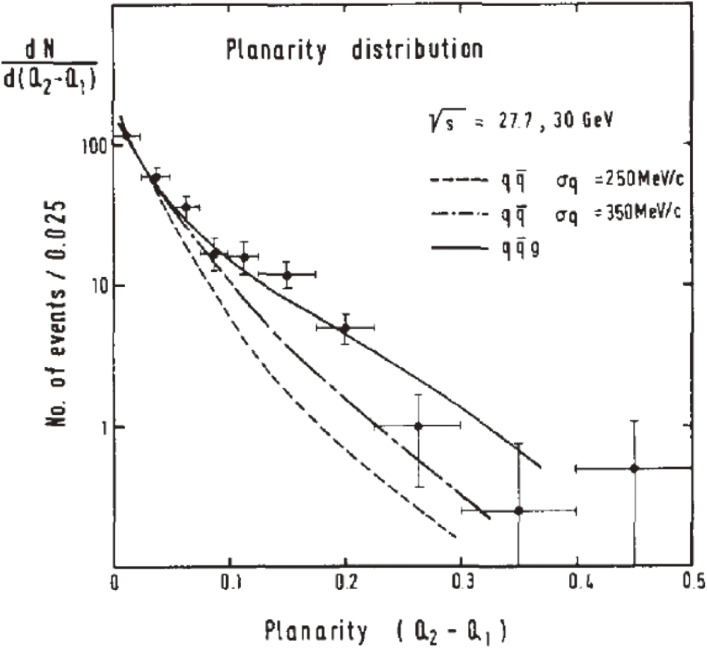
Planarity distribution compared with model predictions.

To see this difference more clearly, projecting the Q-plot onto the planarity (*Q*_2_ − *Q*_1_) axis, which is orthogonal to the sphericity axis, was taken. In Fig. 4, the dotted line indicates a planarity of 0.07. Figure 5 shows the planarity distribution compared with the model predictions. These data show a substantial excess of events in the high planarity region over the $q\bar{q}$ model with σ_*q*_ = 250 MeV. It can also be seen that an arbitrary large value of σ_*q*_ (= 350 MeV) does not account for the excess.

**Figure 6.**
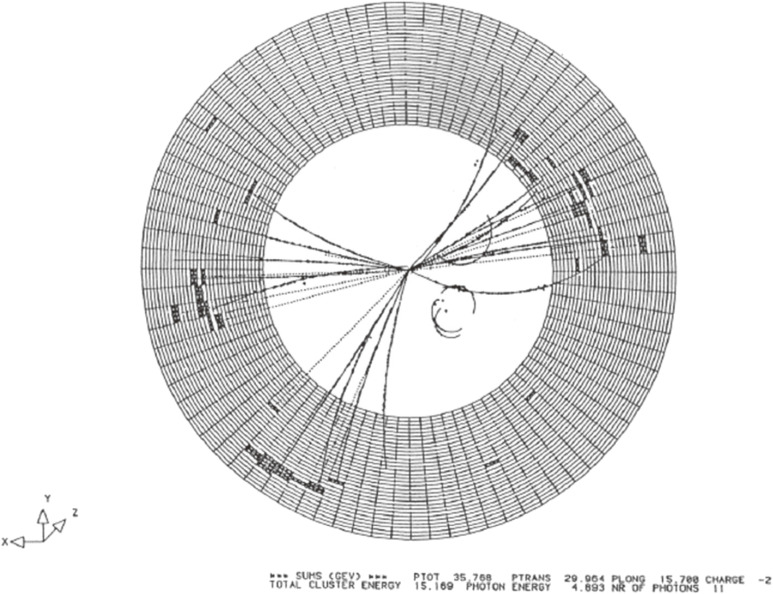
Three-jet event observed by the JADE experiment at PETRA. The view is along the *e*^+^*e*^−^ beam line. The JADE lead-glass shower calorimeter is shown in a perspective, overlaid with charged particle trajectories measured by the inner tracking detector. The shower counters, in which energy had been deposited, are marked in black.

The data agree well with the $q\bar{q}g$ model prediction. The existence of planar events strongly suggests the gluon bremsstrahlung of QCD. As the total energy increases, each jet tends to collimate more sharply, so that some fraction of the planar events should demonstrate a three-jet structure. Figure 6 demonstrates one of such three-jet events taken by JADE experiment. Mark J, TASSO and PLUTO made similar analyses and obtained evidence for the gluon bremsstrahlung as well.^17–19)^

### 2.3 Discovery of weak bosons.

Another big epoch during this period was the discovery of the weak bosons (*W*^±^, *Z*^0^), which came from CERN in 1983.

The validity of the theory of EW interactions had already been proven by various experiments by that time: the discovery of the neutral current by Gargamalle experiment in 1973,^20–22)^ a number of neutrino scattering experiments, atomic parity violation experiments, and the inelastic electron scattering on deuterium at SLAC in 1978.^23)^ These brought Nobel Prizes in Physics 1979 to Sheldon Lee Glashow, Abdus Salam and Steven Weinberg for establishing the theory of the unified weak and electromagnetic interaction.^24–26)^

The EW interaction at the lowest order is described by a single parameter, θ_*W*_, the weak mixing angle, in addition to α and *G*_*F*_. The value of sin^2^ θ_*W*_ had roughly been determined by previous experiments, which predicted the masses of the weak bosons to be *M*_*W*_ ∼ 80 GeV and *M*_*Z*_ ∼ 90 GeV.^27)^

The CERN SPS proton-antiproton collider (Sp$\bar{\text{p}}$S) was designed to provide sufficient collision energy ($\sqrt{s}=540$ GeV) to produce weak bosons. The key element of the Sp$\bar{\text{p}}$S was the technique for the stochastic cooling of antiprotons, invented by Simon van der Meer,^28)^ which made it possible to accumulate enough antiprotons and feed them into the Sp$\bar{\text{p}}$S.

Carlo Rubbia made decisive contributions in initiating the project, and in forming the experiment UA1 to discover the weak bosons. In 1983 the UA1 and UA2 experiments observed clear signals of *W*_±_ productions with the isolated high transverse momentum leptons with the associated missing transverse energy,^29,30)^ followed by a clean peak of *Z*^0^ in the invariant mass distribution of lepton pairs by UA1.^31)^

Carlo Rubbia and Simon van der Meer were jointly awarded Nobel Prizes in Physics 1984.

### 2.4 Candidates of theories beyond the standard model (SM).

It was apparent that the SM should not be the final theory, since it does not include gravity, because it does not explain charge quantization (why the charge of the electron is exactly the same as that of the proton), since it does not explain the particle generation, because it has too many parameters (at least 18), and so on.

Aside from gravity, it is desirable that some kind of theory exists that unifies all of the particles and the interactions in SM; Grand Unified Theory (GUT) to name it. There are a number of choices for the candidate group of GUT, which includes the gauge group of SM; SU(3) × SU(2) × U(1). Among those candidates, the minimum simple group is SU(5), and the next larger group is SO(10).

The minimal SU(5) GUT model covers many shortcomings of SM, and gives new predictions, such as the gauge coupling unification, proton decay, *etc.* However, it soon became apparent that this simple model has a difficulty to explain the long lifetime of the proton from the measurements of Kamiokande, Super-Kamiokande,^32)^ and other nucleon decay experiments.

One possible way out is to go to the larger symmetry group, or to introduce new ideas beyond the SM. One of such ideas is supersymmetry (SUSY), which is a symmetry between bosons and fermions. SUSY is an extension of the space-time symmetries of quantum field theory, which was introduced in particle physics as a possible solution to the gauge hierarchy problem, *i.e.*, to the naturalness or fine-tuning problem of the electroweak scale and the Planck scale against large radiative quantum corrections.^33,34)^ The quadratic divergence appearing in the Higgs boson mass corrections could be eliminated by cancellating the boson and fermion loop contributions. By incorporating SUSY with SU(5) GUT, the proton decay problem could be ameliorated.^35,36)^

The idea of a low-energy SUSY,^33,34)^ being SUSY unbroken above TeV energies, had led to a phenomenological model, Minimal Supersymmetric Standard Model (MSSM). MSSM can also be regarded as being a supersymmetric extension of SM with the minimal particle content. This requires the existence of supersymmetric partner particles corresponding to SM particles. Some early trials to search for new SUSY particles were made based on theoretical predictions,^37–39)^ and a few experimental searches based on those were made in JADE^40–44)^ and in other experiments at PETRA. There was no sign of new particles at the PETRA energies.

Other than SUSY, there was another solution to the gauge hierarchy problem. Since the problem seemed to be related to the large quantum corrections to the Higgs boson mass, the elementary Higgs boson could be replaced by a composite particle, by introducing a new QCD-like theory (“technicolor”) and additional massless fermions (“technifermions”). The technicolor models generate masses for W and Z bosons via dynamical electroweak symmetry breaking mechanism. Those models predict new heavy states, *e.g.*, vector mesons analogous to the ρ and ω mesons in QCD, while there are various possibilities for a composite Higgs boson. Experimental searches for those new states were carried out at higher energy machines.

### 2.5 Summary of this period.

After the “November Revolution”, *i.e.*, the discovery of the c quark, the discoveries of the τ lepton and b quark followed until 1978. During the PETRA period (between 1978 and 1988) new vector bosons (gluon, W and Z) were discovered, which were the first concrete proofs of the validity of the QCD and EW theories.

Tables 3 and 4 show the particle content of SM, fermions and bosons, respectively, which were discovered before 1989, where the particles in hatched area were yet to be found.

In parallel with the experimental verification of SM, new theoretical models were proposed in order to cure the gauge hierarchy problem of SM and to aim for a GUT. One idea was to use SUSY, with which the Higgs boson remains to be an elementary particle, and its mass problem could be cured by canceling the boson and fermion loop contributions. Another idea was to regard the Higgs boson as being a composite particle, and to introduce a dynamical symmetry-breaking mechanism to generate the masses of W and Z. There were no experimental signs during this period for any predictions from those new theories beyond the SM.

## 3 LEP period (1989–)

LEP (Large Electron-Positron Collider) was constructed at CERN, and started its operation in 1989. Until 1995, LEP ran at energies of around the *Z*^0^ peak, *i.e.*, $\sqrt{s}∼91$ GeV (LEP1). From the end of 1995 the *e*^+^*e*^−^ collision energy was increased, reaching 161 GeV in 1996, surpassing the *W*^+^*W*^−^ threshold. LEP continued working until 2000 above the *W*^+^*W*^−^ threshold (LEP2). Its attained highest collision energy was 209 GeV.

**Figure 7.**
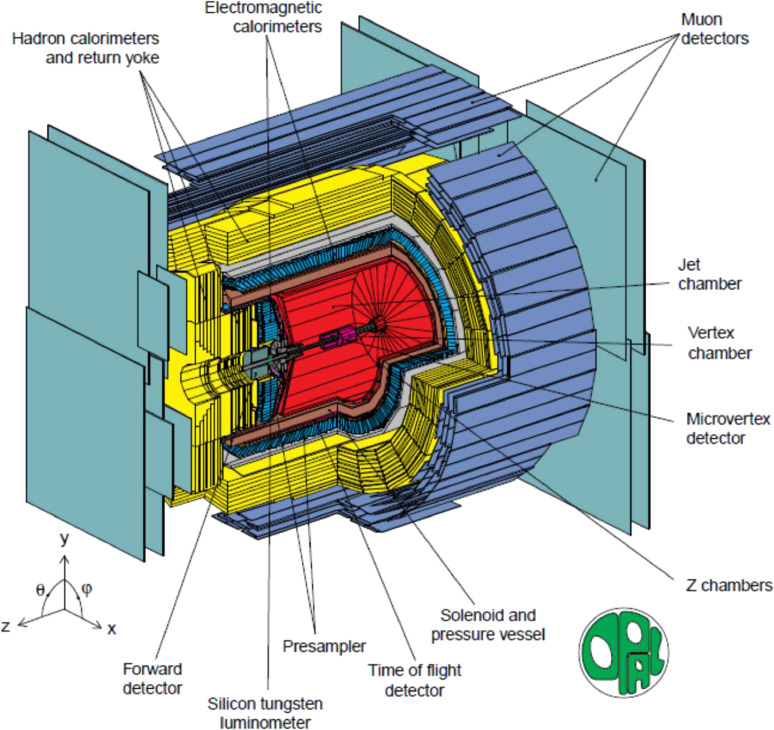
(Color online) General layout of the OPAL detector.

There were 4 experiments at LEP: OPAL, ALEPH, DELPHI and L3. The general layout of the OPAL detector is shown in Fig. 7.^45)^ The ICEPP team was involved in the OPAL experiment and contributed to the construction and operation of barrel part of the electro-magnetic shower calorimeter, which consisted of 9440 lead glass counters.^45–47)^

**Figure 8.**
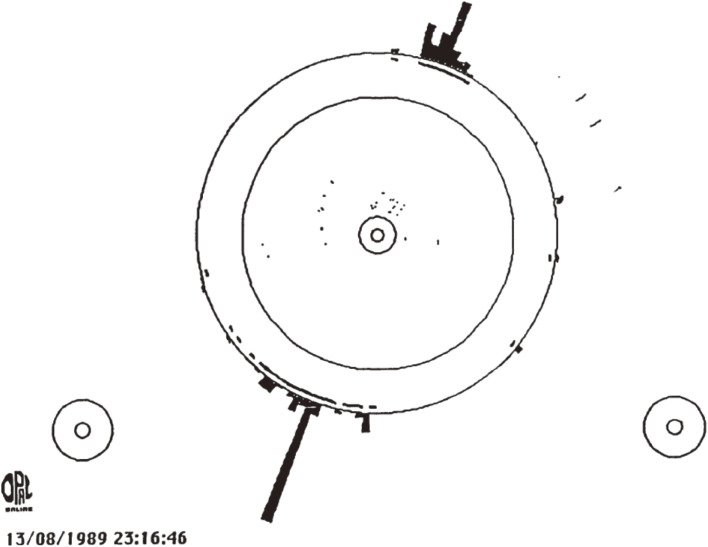
First event at LEP detected by OPAL experiment. The histogram represents the energy deposit in the electro-magnetic shower calorimeter. The inner tracking detector of OPAL was not switched on at that moment.

This calorimeter played an essential role in detecting and identifying the first event at LEP, as shown in Fig. 8, where the histogram represents the energy deposit in the lead glass counters. All of those lead glass counters worked well (with no dead counters) during the entire running period of LEP, and made indispensable contributions to the high-quality data analysis of OPAL due to the hermetic counter arrangement and the stability of the counter operation.

### 3.1 Particle generations.

The first result from the LEP experiments was determining the number of particle generations, *i.e.*, the number of light neutrino species, *N*_ν_. As the SM theory assumes that the neutrinos are massless, *N*_ν_ can be determined from line-shape measurements of the *Z*^0^ resonance.

**Figure 9.**
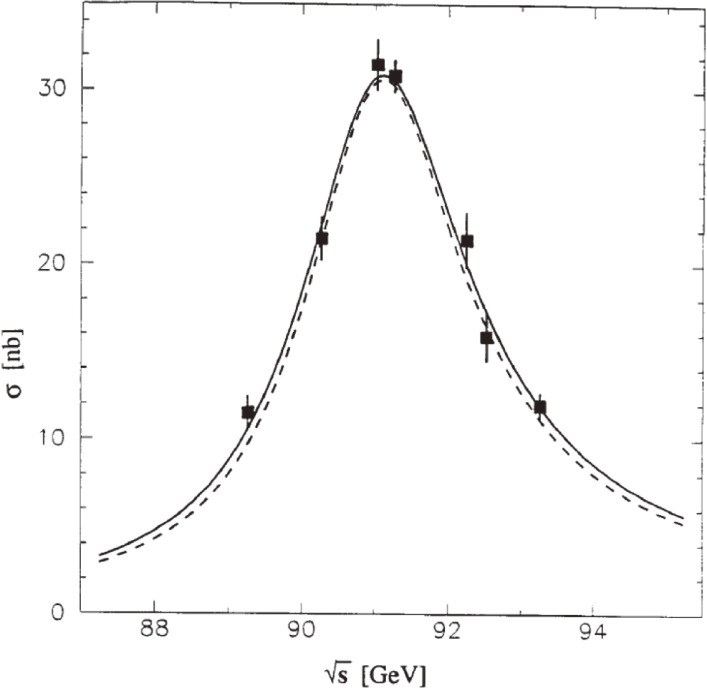
Multihadron cross section versus the center of mass energy measured by the OPAL experiment. The solid curve is a 3-parameter model independent fit. The dashed curve is the best fit from SM, where only *m*_*Z*_ is varied.

The OPAL experiment measured the multihadron cross sections at various c.m. energies at around the *Z*^0^ peak (Fig. 9). In order to determine the mass (*m*_*Z*_) and width (Γ_*Z*_) of *Z*^0^, a Breit-Wigner line shape with *s*-dependent width was used:

$$\tilde{\sigma }(s)=\sigma_{\text{had}}^{\text{pole}}\frac{s\Gamma_{Z}^{2}}{(s-m_{Z}^{2}{)}^{2}+(s^{2}/m_{Z}^{2})\Gamma_{Z}^{2}}.$$ [7]

The overall normalization was

$$\sigma_{\text{had}}^{\text{pole}}=\tilde{\sigma }(s=m_{Z}^{2})=\frac{12\pi }{m_{Z}^{2}}\frac{\Gamma_{e}\Gamma_{\text{had}}}{\Gamma_{Z}^{2}}.$$ [8]

The line-shape formula contains 3 free parameters: *m*_*Z*_, Γ_*Z*_ and $\sigma_{\text{had}}^{\text{pole}}$. The solid curve in Fig. 9 shows the fit result by the OPAL experiment, where these 3 parameters were varied independently.^48)^ The dashed curve in Fig. 9 represents the SM fit with 3 particle generations, where the only free parameter is *m*_*Z*_, since the values of Γ_*Z*_ and $\sigma_{\text{had}}^{\text{pole}}$ can be obtained mainly from *m*_*Z*_, with minor corrections from other parameters of SM. The data are in good agreement with the expectation of SM.

In order to extract the number of light neutrino species (*N*_ν_) from the *Z*^0^ line-shape measurement, the following analysis was made. It was assumed that the neutrinos had the SM coupling and that no other new physics was reflected in the line shape. The *Z*^0^ total width is written as

$$\Gamma_{Z}=\Gamma_{Z}^{\text{SM}}+(N_{\nu }-3)\Gamma_{\nu }^{\text{SM}},$$ [9]

where $\Gamma_{Z}^{SM}$ is the SM value of the total decay width, and $\Gamma_{\nu }^{SM}$ is the SM value of the partial decay width into a single neutrino species. The quantities *m*_*Z*_ and *N*_ν_ were varied, while $\sigma_{\text{had}}^{\text{pole}}$ was fixed by the relation given in.^8)^ The obtained fit result was *N*_ν_ = 3.12 ± 0.42.

Similar analyses were made by the other LEP experiments, and similar results were obtained.^49–51)^ These measurements made a drastic improvement on the previous determinations of *N*_ν_ from Sp$\bar{\text{p}}$S experiments, from PETRA, based on cosmological or astrophysical arguments. The number of particle generations was conclusively determined to be 3.

### 3.2 Precision EW measurements.

Since the LEP could produce tens of millions of *Z*^0^ bosons, it became a very good test bed for the EW theory of SM. During the LEP1 period 4 LEP experiments collected 17 million *Z*^0^ decays in total. The SM expects the *Z*^0^ boson to decay into all species of fermion-pairs, which are kinematically allowed, with similar probability.

Like other experiments at LEP, OPAL made a variety of analyses on the *Z*^0^ resonance. Following the first line-shape result (*e*^+^*e*^−^ → hadrons), several analyses on the leptonic decays of *Z*^0^ and combined analyses of the hadronic and letonic decay channels were performed, obtaining precise *Z*^0^ line shape parameters and EW couplings of charged leptons.^52–55)^

**Figure 10.**
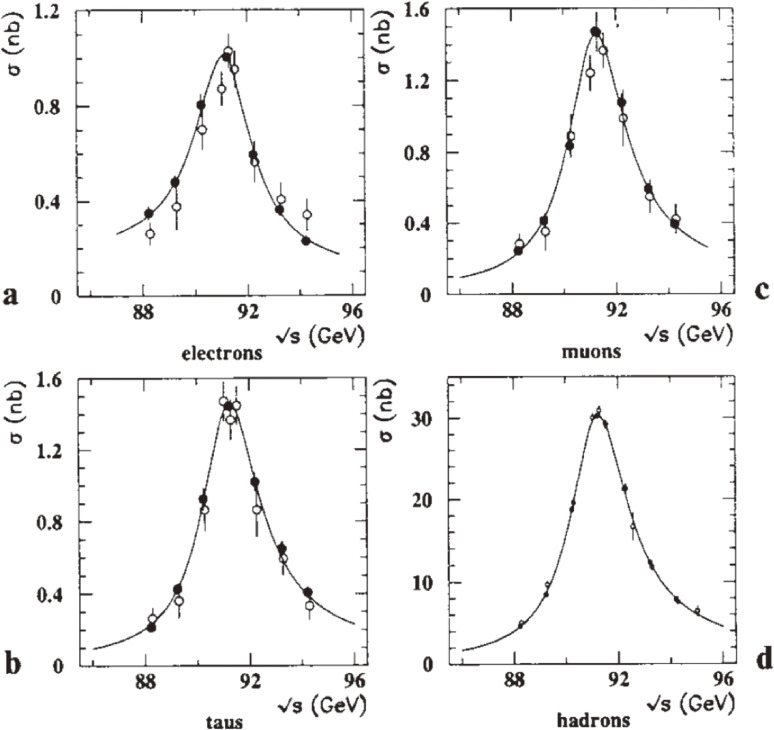
Cross sections as functions of the center-of-mass energy for: **a** *e*^+^*e*^−^ → *e*^+^*e*^−^ with $|\cos\theta_{e^{-}}|<0.7$; **b** *e*^+^*e*^−^ → μ^+^μ^−^; **c** *e*^+^*e*^−^ → τ^+^τ^−^; **d** *e*^+^*e*^−^ → hadrons. The solid lines are the combined fit results. The open and solid points show the 1989 and 1990 data respectively.

**Figure 11.**
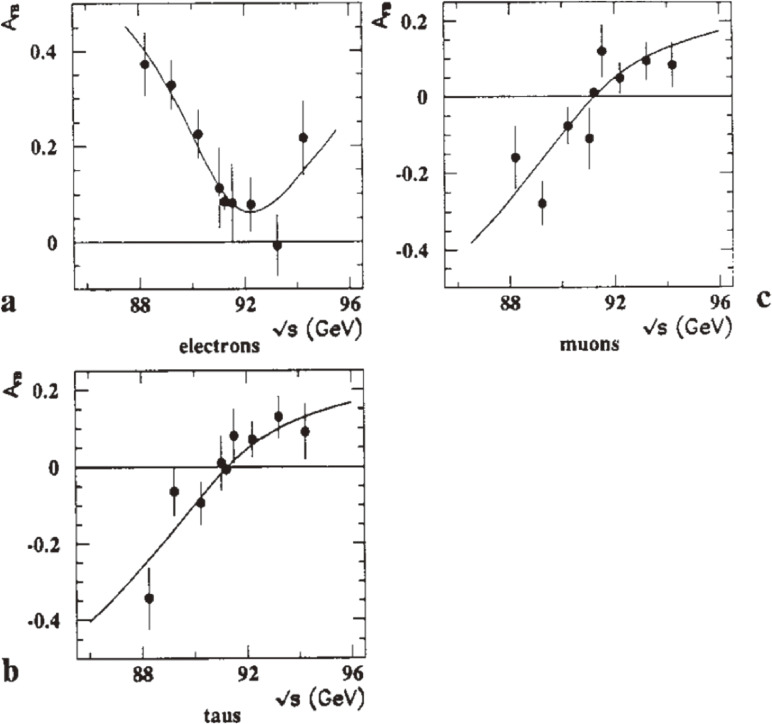
Forward-backward charge asymmetries as functions of the center-of-mass energy for: **a** *e*^+^*e*^−^ → *e*^+^*e*^−^ with $|\cos\theta_{e^{-}}|<0.7$; **b** *e*^+^*e*^−^ → μ^+^μ^−^ with |cos θ| < 0.95; **c** *e*^+^*e*^−^ → τ^+^τ^−^ with |cos θ| < 0.9. The solid lines are the combined fit results. The solid points show the 1990 data.

Figures 10 and 11 show the cross sections and forward-backward charge asymmetries, respectively, as functions of $\sqrt{s}$ for *e*^+^*e*^−^ → lepton pairs and *e*^+^*e*^−^ → hadrons, based on the data taken in 1989 and 1990.^55)^ The forward-backward charge asymmetries were evaluated by counting the numbers of events in the forward and backward polar angular regions, *N*_*F*_ and *N*_*B*_, and using the definition

$$A_{FB}=\frac{N_{F}-N_{B}}{N_{F}+N_{B}}.$$ [10]

In the SM at the tree level, the following relations between the weak and electromagnetic couplings are given:

$$G_{F}=\frac{\pi \alpha }{\sqrt{2}}\frac{1}{m_{W}^{2}\sin^{2}\theta_{W}},$$ [11]

$$\sin^{2}\theta_{W}=1-\frac{m_{W}^{2}}{m_{Z}^{2}},$$ [12]

$$m_{W}^{2}=\frac{m_{Z}^{2}}{2}\left(1+\sqrt{1-\frac{4A}{m_{Z}^{2}}}\right),$$ [13]

with

$$A=\frac{\pi \alpha }{\sqrt{2}G_{F}}\approx (37.28\;\text{GeV}{)}^{2},$$ [14]

where *G*_*F*_ is the Fermi constant, α is the electromagnetic fine-structure constant, *m*_*W*_ is the W boson mass, and θ_*W*_ is the weak mixing angle.

These tree level relations are modified by including the radiative corrections, depending on a chosen renormalization scheme. In the on-shell renormalization scheme Eq. [12] remains valid in higher orders, whereas Eq. [11] is modified as

$$m_{W}^{2}\sin^{2}\theta_{W}=\frac{A}{1-\Delta r}.$$ [15]

The radiative correction, Δ*r*, depends on all of the parameters of SM, particularly the masses of the top quark, *m*_*t*_, and the Higgs boson, *m*_*H*_.

The weak mixing angle, θ_*W*_, derived from Eq. [15] is different from the effective mixing angle, θ_*eff*_, in the neutral-current couplings at the *Z*^0^ peak. The vector and axial-vector couplings of *Z*^0^ to fermion pairs ($f\bar{f}$), *g*_*Vf*_ and *g*_*Af*_ respectively, are defined as

$$g_{Vf}\equiv \sqrt{\rho_{f}}(T_{3}^{f}-2Q_{f}\sin^{2}\theta_{\text{eff}}^{f}),$$ [16]

$$g_{Af}\equiv \sqrt{\rho_{f}}T_{3}^{f},$$ [17]

where $T_{3}^{f}$ is the third component of the weak-isospin, and *Q*_*f*_ is the fermion charge. The relation between these two mixing angles is expressed as

$$\sin^{2}\theta_{\text{eff}}^{f}\equiv \kappa_{f}\sin^{2}\theta_{W},$$ [18]

where κ_*f*_ is a factor representing the difference in the two renormalization schemes.

In order to analyze the cross sections and forward-backward asymmetries (Figs. 10 and 11), OPAL used the following form of the leptonic differential cross section in the improved Born Approximation:

$$\begin{array}{rl} & \frac{2s}{\pi \alpha^{2}}\frac{d\sigma }{d\cos\theta }(e^{+}e^{-}\to l^{+}l^{-})={\left(\frac{1}{1-\Delta \alpha }\right)}^{2}(1+\cos^{2}\theta )\\ & \;+\frac{2Re\chi (s)}{1-\Delta \alpha }[{\hat{v}}_{l}^{2}(1+\cos^{2}\theta )+2{\hat{a}}_{l}^{2}\cos\theta ]\\ & \;+|\chi (s){|}^{2}[({\hat{a}}_{l}^{2}+{\hat{v}}_{l}^{2}{)}^{2}(1+\cos^{2}\theta )+8{\hat{a}}_{l}^{2}\;{\hat{v}}_{l}^{2}\cos\theta ], \end{array}$$ [19]

with

$$\chi =\frac{G_{F}m_{Z}^{2}}{8\pi \alpha \sqrt{2}}\frac{s}{s-m_{Z}^{2}+is\Gamma_{Z}/m_{Z}},$$ [20]

where Δα is the QED vacuum polarization correction, and ${\hat{v}}_{l}$ and ${\hat{a}}_{l}$ are the effective vector and axial-vector couplings:

$${\hat{v}}_{l}^{2}\equiv 4g_{Vl}^{2}=\rho_{l}(1-4\sin^{2}\theta_{\text{eff}}^{l}),$$ [21]

$${\hat{a}}_{l}^{2}\equiv 4g_{Al}^{2}=\rho_{l}.$$ [22]

The main results of the combined fits were the following^55)^:

$$\begin{array}{rl} & N_{\nu }=3.05\pm 0.09,\\ & \rho_{l}=0.998\pm 0.009,\\ & \sin^{2}\theta_{\text{eff}}^{l}=0.238_{-0.006}^{+0.030}. \end{array}$$

Since the top quark mass dependence on the radiative corrections is dominated by the $m_{t}^{2}$ term, while the Higgs mass dependence is logarithmic, a constraint on *m*_*t*_ could be obtained:

$$m_{t}<\text{218\textbackslash{},GeV at 95\% confidence level}.$$

The results of all four experiments at LEP, corresponding to ∼650 thousand *Z*^0^ decays into hadrons and charged leptons collected in 1989 and 1990, were combined to obtain the *Z*^0^ parameters.^56)^ The combined results were:

$$\begin{array}{rl} & N_{\nu }=3.00\pm 0.05,\\ & \sin^{2}\theta_{\text{eff}}^{l}=0.2337\pm 0.0014,\\ & m_{t}=124_{-56-21}^{+40+21}\;\text{GeV}, \end{array}$$ [23]

where the second errors on *m*_*t*_ were systematic errors coming from the variation of *m*_*H*_ in the range of 50–1000 GeV.

In 1994 the CDF experiment at Fermilab announced discovering the top quark produced in $p\bar{p}$ collisions at $\sqrt{s}=1.8$ TeV using the Tevatron.^57)^ By assuming that the top quark decays into W boson and a b jet, the top quark mass was estimated to be $m_{t}=174\pm 10_{-12}^{+13}$ GeV, which was in good agreement with the indirect measurement^23)^ by LEP experiments. This was the first clear evidence for the validity of the EW theory in the higher orders.

The four LEP experiments gathered 17 million Z decays in the LEP1 period, while at SLAC the SLD experiment collected 600 thousand Z decays using a polarized beam at SLC. The LEP experiments measured the cross sections, forward-backward asymmetries (*l*^+^*l*^−^, $q\bar{q}$, $b\bar{b}$, $c\bar{c}$) and τ polarization. The SLD measured the left-right asymmetry:

$$A_{\text{LR}}=\frac{N_{L}-N_{R}}{N_{L}+N_{R}}\frac{1}{\left\langle P_{e}\right\rangle },$$ [24]

where *N*_*L*_ and *N*_*R*_ are the numbers of Z bosons produced by left and right longitudinally polarized electron beams, respectively. $\left\langle P_{e}\right\rangle$ is the luminosity-weighted *e*^−^ beam polarization magnitude, while the *e*^+^ beam at SLC was not polarized. The combination of all these results yielded precise determinations of the *Z*^0^ parameters^58)^:

$$\begin{array}{rl} & m_{Z}=91.1875\pm 0.0021\;\text{GeV},\\ & \Gamma_{Z}=2.4952\pm 0.0023\;\text{GeV},\\ & B(Z\to \text{had})=69.911\pm 0.057\%,\\ & B(Z\to l^{+}l^{-})=3.3658\pm 0.0023\%,\\ & \rho_{l}=1.0050\pm 0.0010,\\ & \sin^{2}\theta_{\text{eff}}^{l}=0.23153\pm 0.00016,\\ & N_{\nu }=2.9840\pm 0.0082, \end{array}$$

where *B*(*Z* → had) is the Z branching fraction into hadrons, and *B*(*Z* → *l*^+^*l*^−^) is the Z branching fraction into chaged lepton pairs of single species assuming the lepton universality.

The four LEP experiments performed measurements at LEP2, collecting 3 fb^−1^ of the integrated luminosity in total, so that the precise EW studies were made on W boson pair production. By combining the results of the four experiments, the fundamental properties of the W boson were obtained.^59)^ In particular, the mass and width of W, *m*_*W*_ and Γ_*W*_, and the branching fraction of W decays to hadrons, *B*(*W* → had), were determined to be:

$$\begin{array}{rl} & m_{W}=80.376\pm 0.033\;\text{GeV},\\ & \Gamma_{W}=2.195\pm 0.083\;\text{GeV},\\ & B(W\to \text{had})=67.41\pm 0.27\%. \end{array}$$

**Figure 12.**
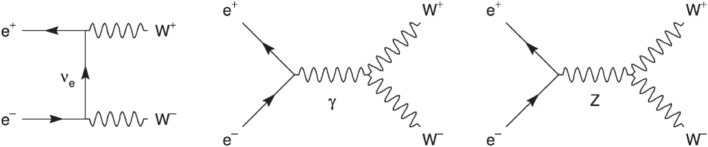
Feynman diagrams for the process *e*^+^*e*^−^ → *W*^+^*W*^−^ at the tree level.

According to the SM, W bosons are pair-produced via the processes at the tree level, as shown in Fig. 12, involving t-channel ν_*e*_ exchange and s-channel γ and Z exchange. The s-channel diagrams, *i.e.*, the triple gauge couplings, manifest the non-Abelian nature of the SU(2) × U(1) gauge theory. Its direct study was made possible at LEP2, while at LEP1 and at SLC the couplings of *Z*^0^ to fermion pairs were studied precisely.

**Figure 13.**
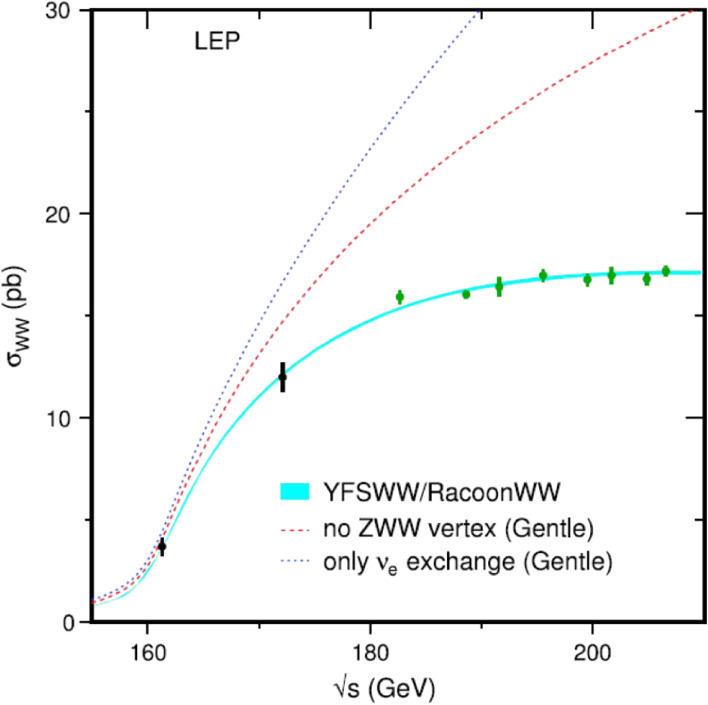
(Color online) LEP measurements of the W-pair production cross section, compared to the theoretical predictions.

Figure 13 shows the combined LEP W-pair production cross section as a function of the center-of-mass energy. The experimental data are compared with the theoretical predictions: 1) with all the diagrams in Fig. 12 (YFSWW/RacoonWW), 2) with no ZWW vertex, and 3) with only the ν_*e*_ exchange diagram. The need for a diagram with a ZWW vertex is apparent, and it is a remarkable confirmation of the non-Abelian nature of the EW theory.

All of these precision measurements proved the validity of the EW theory, not only in the tree level, but also in the quantum correction level. This brought Nobel Prizes in Physics 1999 to Gerardus ’t Hooft and Martinus J.G. Veltman, who proved the renormalizability of the theory.

To go a step further, like the top-quark mass was predicted from the indirect precision measurements, the Higgs boson mass can also be obtained in a similar way. By combining the indirect EW measurements and the direct measurements of *m*_*t*_ and *m*_*W*_, a fit was performed to predict the Higgs-boson mass, *m*_*H*_.^58)^ The obtained result was:

$$m_{H}=129_{-49}^{+74}\;\text{GeV},$$

or *m*_*H*_ < 285 GeV at the 95% confidence level.

### 3.3 Direct search for Higgs boson.

The SM Higgs boson was expected to be directly produced at LEP1 mainly in association with a virtual *Z*^0^ boson (Z*). The search channels at an early stage of LEP1 were therefore *e*^+^*e*^−^ → Z*H^0^, with Z* → (*e*^+^*e*^−^ or μ^+^μ^−^ or $\nu \bar{\nu }$) and *H*^0^ → ($q\bar{q}$ or τ^+^τ^−^).^60)^ By searching *H*^0^ for the whole LEP1 period, OPAL did not find any positive signals, and obtained a mass lower limit of 60 GeV.^61)^ The other LEP experiments obtained similar results.

AT LEP2 the Higgs boson could be produced in association with a real *Z*^0^ boson, where all of the decay channels of *Z*^0^ could be used in the analysis (*Z*^0^ → *e*^+^*e*^−^, μ^+^μ^−^, τ^+^τ^−^, $\nu \bar{\nu }$ and $q\bar{q}$). For the Higgs boson above the LEP1 limit, the main decay channels were *H*^0^ → *b*$\bar{b}$ and *H*^0^ → τ^+^τ^−^. None of the LEP experiments obtained significant signals for *H*^0^ production. The combined results of the four LEP experiments were *m*_*H*_ > 114.4 GeV at the 95% confidence level.^62)^

These results of the direct *H*^0^ search are consistent with the indirect search mentioned in the previous subsection. Within the framework of the SM, the mass of the Higgs boson was constrained by LEP experiments to be in the narrow region: 114.4 GeV < *m*_*H*_ < 285 GeV.

### 3.4 Verification of QCD.

Following analyses of jet productions in *e*^+^*e*^−^ collisions, mostly based on data from PETRA experiments, the abundant LEP collider data on the *Z*^0^ resonance as well as at LEP2 were used for detailed QCD studies.

One of the essential tests of perturbative QCD is “running” of the QCD coupling constant, α_*s*_, which is the only free parameter in the theory when the quarks are treated as being massless. This manifests the asymptotic freedom of QCD, a steady reduction of the strong coupling as the energy scale of the interaction increases.

The LEP measurements of α_*s*_ covered a wide range of energies, from $\sqrt{s}=1.78$ GeV (the mass of τ lepton) to 206 GeV. The hadronic partial decay width of the *Z*^0^, Γ_had_, can be used to extract a precise value of α_*s*_ at $\sqrt{s}=m_{Z}$, since it is one of the most precisely measured quantities, and its QCD corrections are known up to the next-next-to-leading order (NNLO), *i.e.*, to $O(\alpha_{s}^{3})$. The combined result of LEP experiments was:

$$\alpha_{s}(m_{Z})=0.1226_{-0.0038}^{+0.0058},$$

obtained from the ratio $R_{Z}=\Gamma_{\text{had}}/\Gamma_{l^{+}l^{-}}$.^63)^ Similarly, a significant determination of α_*s*_ was made at a small energy scale, obtained from the normalized hadronic branching fraction of τ leptons, *R*_τ_ = Γ(τ → hadrons ν_τ_)/Γ(τ → *e*ν_*e*_ν_τ_):

$$\alpha_{s}(m_{\tau })=0.322\pm 0.005(\text{exp.})\pm 0.030(\text{theo.}).$$

Determinations of α_*s*_ from hadronic event shape observables and from jet production rates were made from LEP1 to LEP2 energies, $\sqrt{s}=91.2$ GeV–206.0 GeV.

**Figure 14.**
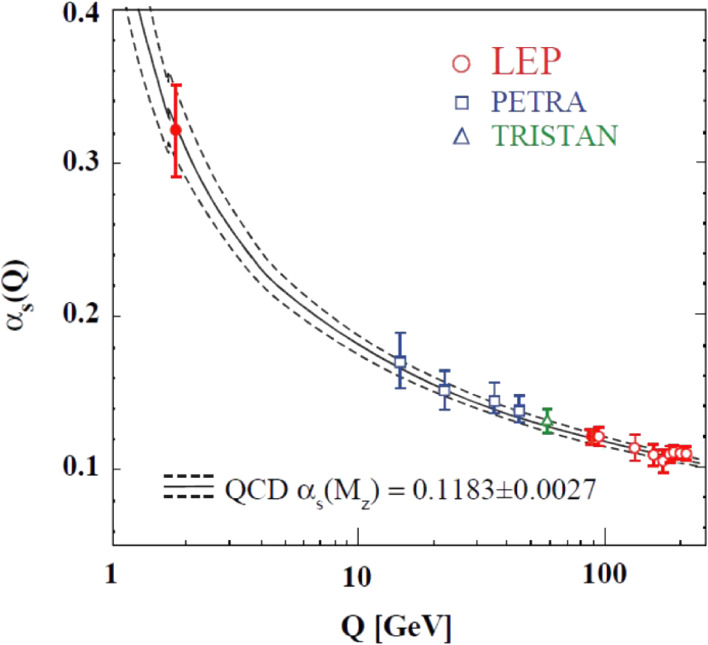
(Color online) Summary of LEP measurements of α_*s*_(*Q*), where *Q*^2^ = *s*. Results from PETRA and TRISTAN experiments are also included. The curves represent the QCD predictions of α_*s*_(*Q*) for the world average of α_*s*_(*m*_*Z*_).

The LEP measurements of α_*s*_ are summarized in Fig. 14,^63)^ together with PETRA^64)^ and TRISTAN^65)^ measurements. The data are compared with the QCD predictions of α_*s*_ for the world average of α_*s*_(*m*_*Z*_) = 0.1183 ± 0.0027. This clearly exhibits that the specific energy dependence of α_*s*_, and hence the concept of asymptotic freedom, are verified by the experimental data.

In 2004 Nobel Prizes in Physics were awarded to David J. Gross, H. David Politzer and Frank Wilczek for discovering asymptotic freedom in the theory of strong interactions.^66–70)^

The experimental investigations of the gauge structure of QCD were further pursued at LEP. The key element giving rise to the asymptotic freedom is the gluon self-coupling, *i.e.*, that gluons can interact with themselves. This was studied in angular correlations and energy distributions of four-jet events.^71,72)^ The results were combined to determine the color factors of QCD; *C*_*A*_ associated with gluon emission from a gluon, and *C*_*F*_ associated with gluon emission from a quark^73)^:

$$\begin{array}{rl} & C_{A}=2.89\pm 0.21,\\ & C_{F}=1.30\pm 0.09, \end{array}$$

which are in excellent agreement with the gauge structure constants of QCD (*C*_*A*_ = 3, *C*_*F*_ = 4/3).

It should be fair to mention here that similar results had been obtained by the TRISTAN experiments by that time, though with a bit less precision. The angular correlations among the jets in the four-jet events were analyzed, and the non-Abelian nature of the QCD was studied. The first result was obtained by AMY,^74)^ excluding the Abelian model of QCD at the 90% C.L., followed by TOPAZ and VENUS,^75)^ raising the exclusion level to 95%.

QCD predicts that quarks and gluons behave differently regarding the fragmentations due to their different color charges. At LEP1 a study was made using hadronic *Z*^0^ decays, and identifying the gluon jets in $b\bar{b}g$ events using the b-tagging method. Various distributions, such as rapidity, energy, transverse momentum with respect to the jet axes, *etc.* were compared for charged particles in light quark and gluon jets. It was observed that the charged-particle multiplicity ratio of gluon to quark jets to be 2.29 ± 0.09 (stat.) ± 0.15 (syst.), in good agreement with the QCD expectation of *C*_*A*_/*C*_*F*_ = 2.25.^76)^

### 3.5 Other discoveries in SM.

There were two more major discoveries in SM from experiments other than the high-energy collider experiments.

Following the discovery of the sixth quark (top) in 1994 at Fermilab, the sixth lepton (tau neutrino, ν_τ_) was discovered in 2000 also at Fermilab by the DONUT experiment.^77)^ They recorded and analyzed the high-energy neutrino interactions in nuclear emulsion targets. After τ decay searches, they observed four ν_τ_ events with an estimated background of 0.34 events, which was consistent with the SM expectation.

This completed the list of all fermions in SM with 3 generations (Table 1). Regarding the bosons (Table 2), all of the vector bosons were made present by this time, while only the scaler boson (the Higgs) was yet to be found.

Another discovery was made at KEK and at SLAC, which observed the CP violation in B-meson decays. The CP violation had first been discovered in K-meson decay. However, it could not been explained until a theory formulated by M. Kobayashi and T. Maskawa in 1973, by introducing 6 quarks within the framework of the EW theory, while there were only 3 quarks known then to exist.^78)^ The Belle experiment at KEKB and the Babar experiment at PEP-II observed clear signals of CP violation in B-meson decays, so that the validity of the Kobayashi-Maskawa theory was proven. Details of the discovery were described by F. Takasaki in Proc. Jpn. Acad., Ser. B published in 2012.^79)^

### 3.6 Search for new physics beyond SM.

At the beginning of the LEP period, there were two main directions for new physics beyond the SM. The key issue was the mechanism for EW symmetry breaking in the SM, which resulted in the question as to whether the Higgs boson is elementary or composite.

The most typical and seemingly quite natural way to keep the Higgs boson as an elementary particle was to introduce SUSY in SM. As it was described in subsection 2.4, MSSM appeared to be very promissing, but no sign of SUSY had been observed.

**Figure 15.**
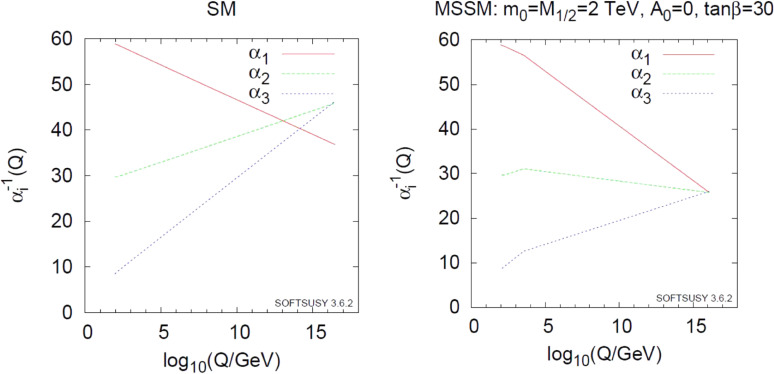
(Color online) Running couplings in SM and MSSM using two-loop renormalization group revolution. This figure was taken from Ref. 81 (2018).

Soon after the LEP experiments started, the collaborators of the DELPHI experiment published an interesting analysis concerning the gauge coupling unification using DELPHI data.^80)^ Their result showed that the GUT prediction of gauge coupling unification, *i.e.*, the tree coupling constants would become equal at a single unification point, did not work in SM. In contrast, the MSSM led to good agreement with a single unification scale of 10^16^ GeV, which was consistent with the limit on the proton lifetime. In addition, their fit result indicated the SUSY scale to be at around 1000 GeV, which gave a strong hope for the SUSY particles to have masses in the TeV region. Figure 15 shows the latest analysis result taken from Ref. 81 (2018).

Direct searches for SUSY particles (scalar leptons, scalar quarks, charginos and neutralinos) were carried out by LEP experiments. OPAL, like other LEP experiments, made searches in the *Z*^0^ decay channels,^82,83)^ and at each energy point of LEP2 up to its highest energies.^84–86)^ None of them showed any sign of direct productions of SUSY particles.

Another direction for new physics beyond the SM was dynamical EW symmetry breaking. In such theories, massive weak bosons acquire masses through the mechanism that their longitudinal components are identified as composite Nambu-Goldstone bosons arising from dynamical symmetry breaking in a strongly-coupled extension of the SM. The first and simplest models of dynamical EW symmetry breaking were technicolor theories,^87,88)^ as described in subsection 2.4.

M.E. Peskin and T. Takeuchi introduced new parameters, S and T, representing the EW radiative corrections, and calculated the contributions from various models of new physics.^89)^ Early precision measurements at LEP and SLC, especially on the value of S, soon excluded a large parameter space of the simple technicolor models,^90,91)^ giving a necessity for theories to incorporate new ideas.

The first window of new physics beyond the SM was opened in the neutrino sector. In the framework of the SM, neutrinos are assumed to be precisely massless. The accumulation of experimental evidence from Kamiokande, Super-Kamiokande, SNO and other solar neutrino experiments brought the discovery of neutrino oscillations, *i.e.*, both atmospheric neutrinos (ν_μ_) and solar neutrinos (ν_*e*_) oscillate with different species of neutrinos. This meant that neutrinos have masses, though they are much lighter than that of other fermions. The search for the origins of the neutrino masses, neutrino mixing parameters and the CP violation in the lepton sector would open up a new field of physics beyond the SM. Details of the discovery of neutrino oscillations were described in Proc. Jpn. Acad., Ser. B by T. Kajita on atmospheric neutrinos (in 2010)^92)^ and by M. Nakahata on solar neutrinos (in 2011).^93)^

### 3.7 Summary of this period.

From the view-point of verifying the SM, this period was very productive and fruitful. Starting from determining the particle generations, high-energy collider experiments produced results that essentially confirmed the validity of the EW theory and QCD at the higher order quantum level with high-precision measurements of various observables.

There were also important discoveries, such as the top quark, tau neutrino, and CP violation in the quark sector. As a result, all of the ingredients of SM were discovered and confirmed, except for the Higgs boson. Whether the Higgs boson exists or not, and whether its properties conform to the SM expectations or not, remained to be the biggest and most urgent questions regarding the next-generation experiments.

In addition to verifying the SM, new observations toward physics beyond the SM appeared during this period. One is the discovery of neutrino oscillations, and therefore non-zero neutrino masses. Another observation was the possibility of low-energy SUSY, which gave a strong motivation for SUSY searches in the next period.

## 4 LHC period (2009–)

The LHC (Large Hadron Collider), proton-proton collider, producing a collision energy ($\sqrt{s}$) of 14 TeV, was constructed at CERN in the LEP tunnel after termination of LEP operation. LHC started operating in September 2008 with the injection of a single beam, but just after 9 days it experienced an incident that damaged a large part of the collider machine. It took more than a year for recovery and restarting its operation in November 2009 with successful collisions at an injection energy of 450 GeV + 450 GeV.

As the restoration could not be fully completed for the design energy of $\sqrt{s}=14$ TeV within a short time of ∼1 year, it was decided to start running at $\sqrt{s}=7$ TeV. In March 2010 LHC succeeded beam collisions at $\sqrt{s}=7$ TeV, and started physics runs.

There are 4 experiments at LHC: ATLAS, CMS, LHCb and ALICE. ATLAS^94)^ and CMS^95)^ are general-purpose detectors aimed at studies of physics at the highest possible energies. LHCb is a specialized detector for physics related to b quark, whereas ALICE is dedicated to heavy-ion (Pb) collision mode of LHC.

The ICEPP team was involved in the ATLAS experiment from its formation of the collaboration in 1992. The ATLAS-Japan group was formed in 1994 by 12 Japanese institutes, including ICEPP, KEK, Kobe University and others, to participate in the ATLAS collaboration. The contributions of the ATLAS-Japan group covered a large area in the ATLAS experiment: a silicon inner tracker, a solenoidal magnet, a forward muon trigger detector, a trigger/data acquisition system as well as software with Geant4, and Tier-2 data analysis center for the worldwide LHC computing GRID system.

### 4.1 Confirmation of SM.

From the start of LHC beam collisions at $\sqrt{s}=900$ GeV in 2009, followed by high-energy runs at $\sqrt{s}=7$ TeV in 2010 and in 2011, LHC experiments successfully accumulated sufficient data to confirm the performance of the detectors, and to tune the simulation software.

Since the proton is not a point-like particle, it is necessary to know its structure functions in order to analyze *pp* collision processes. In high-energy *pp* collisions they are expressed in the parton distribution functions (PDFs), which had been precisely determined by the previous fixed-target experiments and collider experiments (Tevatron and HERA), as well as by the LHC experiments themselves.^96)^

**Figure 16.**
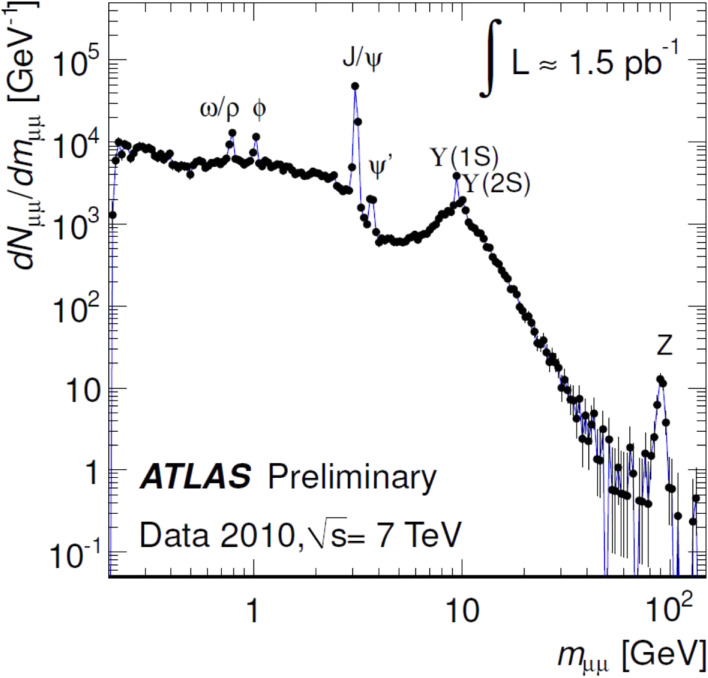
(Color online) Reconstructed invariant mass distribution of opposite-sign muon pairs. The events were selected in the first data sample at $\sqrt{s}=7$ TeV taken in 2010 corresponding to the integrated luminosity of 1.5 pb^−1^.

One of the plots showing how quickly the ATLAS (and the CMS too) could calibrate the data and tune the necessary software for analyses is given in Fig. 16. It shows the reconstructed invariant mass distribution of opposite-sign muon pairs, in which the events were selected in the first data sample at $\sqrt{s}=7$ TeV, taken in 2010, corresponding to an integrated luminosity of 1.5 pb^−1^. All of the relevant SM particles can be clearly seen, which shows the power of lepton identification in the high-energy hadron collider environment, and that the experiment is ready for physics studies.

Muon analyses, as well as electron analyses, were elaborated using whole data in 2010, corresponding to an integrated luminosity of 40 pb^−1^,^97)^ and applied to studied of the Drell-Yan process, *i.e.*, dilepton (*l*^+^*l*^−^) production via the s-channel exchange of a virtual photon or Z boson in pp collisions, based on the data of 2010 and 2011. The Drell-Yan process has a small experimental uncertainty and low backgrounds, allowing for a precision test of SM, as well as providing important information on the partonic structure of the proton. ATLAS allowed for analyses in the low invariant mass region (between 26 GeV and 66 GeV), including a part of data collected in 2011 with an integrated luminosity of 1.6 fb^−1^,^98)^ and in the high invariant mass region (between 116 GeV and 1500 GeV) based on the full 2011 data set with an integrated luminosity of 4.9 fb^−1^.^99)^ Both results were consistent with the SM expectations.

By extending the lepton analyses, ATLAS (and also CMS) conducted measurements concerning inclusive W boson production,^100)^ as well as diboson (*W*^+^*W*^−^, ZZ and WZ) productions.^101–103)^ Further, by including hadronic jet informations, top-quark pair production could be studied, in which the top quark decays as *t* → *bW*.^104)^ All of these processes were in good agreement with predictions of the SM.

### 4.2 Discovery of Higgs boson.

In 2012 LHC increased the collision energy to $\sqrt{s}=8$ TeV, and started physics runs in April. By June, LHC had delivered to each experiment more than 5 fb^−1^, *i.e.*, a similar amount as in the previous year’s run at $\sqrt{s}=7$ TeV. Both ATLAS and CMS analyzed those data, and in July they announced the discovery of a new particle with its mass at around 125 GeV, which is consistent with the SM Higgs boson.^105,106)^

**Figure 17.**
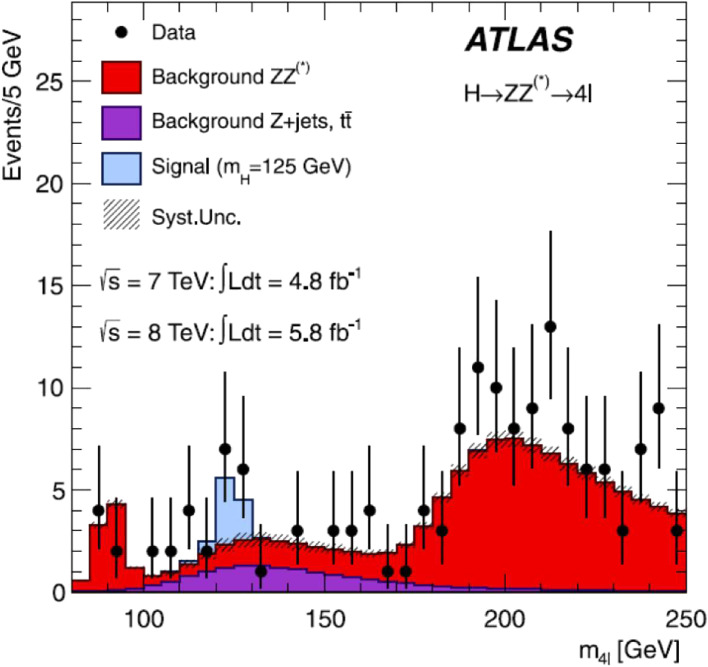
(Color online) Distribution of the 4-lepton invariant mass measured by the ATLAS experiment, compared to the SM background expectation. The signal expectation for an SM Higgs boson with *m*_*H*_ = 125 GeV is also shown.

The search for the SM Higgs boson through the decay channel *H* → *ZZ*^(^*^)^ → 4*l*, where *l* = *e*, μ, provides good sensitivity over a wide mass range (110–600 GeV), due to the excellent momentum resolution of the ATLAS (and also CMS) detector. Figure 17 shows the distribution of the 4-lepton invariant mass measured by the ATLAS experiment, compared to the SM background expectation. The signal expectation for an SM Higgs boson with *m*_*H*_ = 125 GeV is also shown.

The major backgrounds come from continuum productions of *ZZ*^(^*^)^ (including *Z*^(^*^)^γ* and γ*γ*), *Z* + jets and $t\bar{t}$. The excess of events observed near *m*_4__*l*_ = 125 GeV has a local maximum value of the significance, reaching 3.6σ.

**Figure 18.**
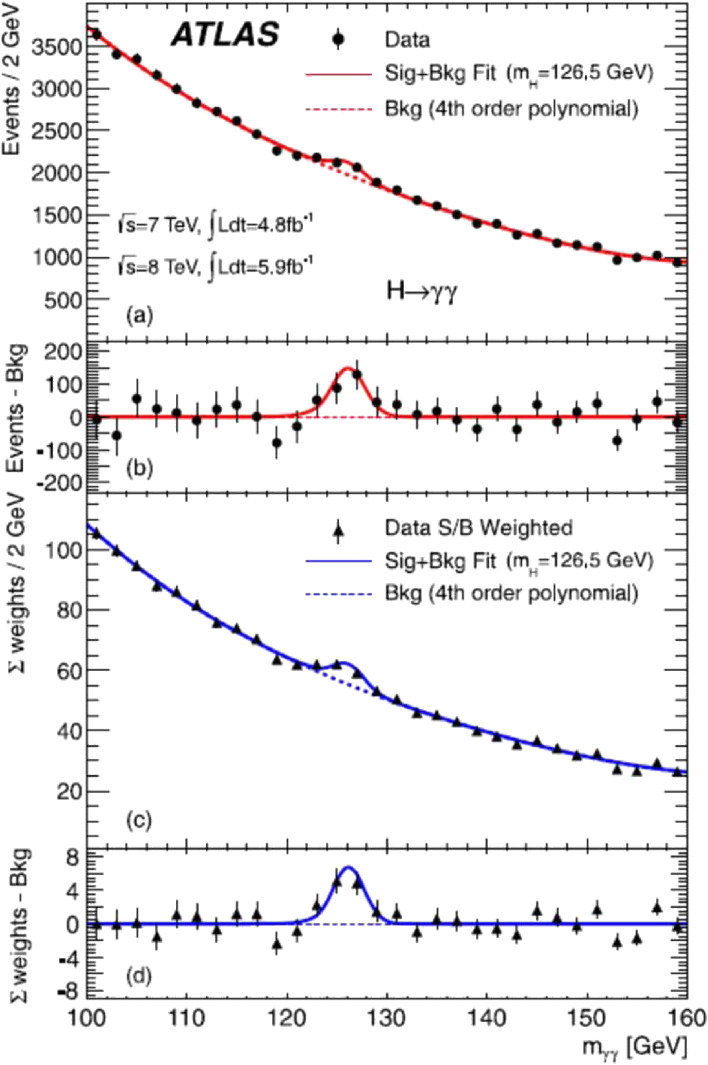
(Color online) Distributions of the diphoton invariant mass measured by the ATLAS experiment. The inclusive sample is shown in (a) and a weighted version of the same sample in (c). The results of a fit to the data with a signal component fixed to *m*_*H*_ = 126.5 GeV and a background component described by a fourth-order polynomial is superimposed. The residual of the data and weighted data with respect to the respective fitted background component are displayed in (b) and (d).

A search for the SM Higgs boson was also performed through the decay channel *H* → γγ in the mass range between 110 GeV and 150 GeV. Figure 18 shows the distributions of the diphoton invariant mass measured by the ATLAS experiment.

The dominant background is SM diphoton production (γγ). The contributions also come from γ + jet and jet + jet production with one or two jets mis-identified as photons, and from the Drell-Yan process. An inclusive sample is shown in Fig. 18(a). The results of a fit to the data with a signal component fixed to *m*_*H*_ = 126.5 GeV and a background component described by a fourth-order polynomial is superimposed. The residual of the data with respect to the respective fitted background component is displayed in Fig. 18(b).

In order to increase the sensitivity to a Higgs boson signal, the events are separated into ten exclusive categories of different *p*_*T*_ and rapidity regions, *etc.*, each having different mass resolutions and signal-to-background ratios. A statistical analysis of the data employs an unbinned likelihood function constructed from those of the ten categories of the data sample. Figure 18(c) shows the mass spectrum obtained after weighting events with category-dependent factors reflecting the signal-to-background ratios; Figure 18(d) shows the residual of data with respect to the fitted background component.

The excess of events observed near to *m*_4__*l*_ = 126.5 GeV has a local maximum value of the significance reaching 4.5σ.

The decay channel *H* → *WW*^(^*^)^ → *e*νμν, with two opposite-charge leptons, has also been used for the SM Higgs boson search, though it has a wider mass distribution. The significance value at around *m*_*H*_ = 125 GeV was found to be 2.8σ.

The combined results (*H* → *ZZ*^(^*^)^ → 4*l*, *H* → γγ and *H* → *WW*^(^*^)^ → *e*νμν) show clear evidence for a neutral boson with a measured mass of 126.0 ± 0.4 (stat.) ± 0.4 (sys.) GeV, which has a significance of 5.9σ, corresponding to a background fluctuation probability of 1.7 × 10^−9^. It is also compatible with the production and decay of the SM Higgs boson. CMS made a similar analysis, and also obtained a clear excess of events with a local significance of 5.0 σ at a mass of 125.3 ± 0.4 (stat.) ± 0.5 (sys.) GeV, LHC supplied ∼20 fb^−1^ to each experiment in 2012. ATLAS and CMS made further studies of the spin and parity quantum numbers of the Higgs boson using the whole dataset taken so far.^107–109)^

The SM Higgs boson is assumed to have a spin-parity (*J*^*P*^) 0^+^. This hypothesis was compared to an alternative hypotheses with *J*^*P*^ = 0^−^, 1^+^, 1^−^, 2^+^, based on the kinematic properties of the decay channels *H* → *ZZ*^(^*^)^ → 4*l*, *H* → γγ and *H* → *WW*^(^*^)^ → *e*νμν. These studies provided strong evidence for the scalar (0^+^) nature of the Higgs boson.

In 2013 Nobel Prizes in Physics were awarded to François Englert^110)^ and Peter Higgs^111)^ “for the theoretical discovery of a mechanism that contributes to our understanding of the origin of mass of subatomic particles, and which was recently confirmed through the discovery of the predicted fundamental particle, by the ATLAS and CMS experiments at CERN’s Large Hadron Collider”.

After Run 1, from 2009 to 2012, LHC raised the collision energy to $\sqrt{s}=13$ TeV in Run 2, from 2015 to 2018, and delivered to each of the ATLAS and CMS experiments an integrated luminosity of over 150 fb^−1^.

In Run 1, studies on the Higgs boson were made mainly through channels related to couplings of the Higgs boson to the vector gauge bosons (*W*^±^, *Z* and γ). The excellent performance of LHC Run 2 and experiments made it possible to study the Yakawa couplings of the Higgs boson, *i.e.*, the couplings to the charged fermions of the 3-rd generation (*t*, *b* and τ).^112)^

So far, the experimental measurements of all the observed production and decay channels of the Higgs boson are consistent with the SM predictions. However, to study the couplings of the Higgs boson to lighter fermions or the Higgs self-couplings, for example, more data are needed.

### 4.3 Directions toward new physics beyond the SM.

The long exploration to verify the standard model of particle physics came to an end with the discovery of the Higgs boson and its various confirmations. However, as described in subsections 2.4 and 3.6, there should be a new physics beyond the SM.

SUSY was the prime candidate among such new physics. However, there are still no signs of SUSY particles, after LHC Run 2, in the mass region of up to 1–2 TeV.^112)^ Although the results depend on assuming various SUSY models, and the limits can be much weaker, models of the low-energy SUSY would need some modifications and/or new ideas.

By avoiding difficulties of the technicolor models with the experimental results, many theories of dynamical EW symmetry breaking were developed. Most of such theories predict the existence of new resonances in the TeV region. No significant signs have been observed so far by LHC Run 2.^112)^

If quarks and leptons are composite particles and the composite scale is TeV, or slightly above, the effect of the new interactions should be seen at LHC. One example is the scattering cross section for *qq* → *qq*, which might differ from the SM predictions. Another example is the appearance of excited quarks and leptons, of which the signals are the narrow resonance peaks. After LHC Run 2, no significant deviations from the SM have been observed.^112)^

Very interesting new theoretical models emerged, several years after LHC was decided to be built, to solve the gauge hierarchy problem, which does not use SUSY nor technicolor. The theoretical framework uses the idea of extra spacial dimensions, which is required to describe a consistent theory of quantum gravity, *i.e.*, superstring theory.

The first model assumes that the extra dimensions can be as large as a millimeter scale, while in the original superstring theories the extra dimensions are supposed be compactified at a scale close to the Planck scale.^113)^ Another model uses a warped geometry that postulates the compactification scale of the extra dimension to be on the order of 1/TeV.^114)^

Both models, as well as many varieties of extra-dimension models appeared later, predict that the gravitational force would give sizable effects at the LHC energy region. In some particular cases LHC can even produce mini-black holes. So far, no signatures for mini-black hole productions have been seen at LHC, nor any significant deviations in the available distributions from predictions of the SM.^112)^

### 4.4 Limit of SM.

If there are no new physics beyond the SM at around the TeV region, or above, then the question arises as to where the SM remains valid.

In the SM a complex scaler Higgs doublet, $\phi \equiv \left(\begin{array}{c} \phi^{+}\\ \phi^{0} \end{array}\right)$, is added to give masses to the weak vector bosons and to fermions through spontaneous symmetry breaking with the potential given by

$$V(\phi )=\mu^{2}\phi^{†}\phi +\lambda (\phi^{†}\phi {)}^{2}.$$ [25]

Here, μ, which is directly related to the Higgs boson mass, and λ, the quartic coupling, are free parameters in the theory.

In calculating higher order radiative corrections, the vector boson part and the fermion part are protected from the divergence problem by the gauge symmetry and the chiral symmetry, respectively. However, the scalar (*i.e.*, Higgs) part has no symmetry in the SM to protect from the divergence, although SUSY is a nice candidate for this problem. Hence the quartic coupling could become non-perturbative, or negative, which implies that the Higgs vacuum would become unstable. If either of these cases occurs below the Planck scale, it means that there should exist a new physics beyond the SM below that energy scale.

Radiative corrections of the quartic coupling, λ, depend strongly on the values of the Higgs boson mass (*m*_*H*_) and the top quark mass (*m*_*t*_). The experimentally measured values of *m*_*H*_ and *m*_*t*_ indicate that the EW vacuum of the Higgs potential is most likely metastable, *i.e.*, the high-energy evolution of λ shows that it becomes negative at energies of around Λ=O(10^10^–10^12^) GeV.^112)^ However, if *m*_*t*_ differs from the currently measured value by 3σ, λ could remain positive all the way to the Planck scale, which might open very interesting possibilities, such as Higgs inflation in which the SM Higgs boson plays the role of “inflation”, and so on.

Thus the higher precision measurements of *m*_*H*_ and *m*_*t*_, as well as exploring the detailed nature of the Higgs boson and the mechanism of EW symmetry breaking, are very important, since these would open a window towards the Planck scale.

## 5 Summary and outlook for future

For about 40 years after 1974, all ingredients of the standard model for particle physics have been discovered and verified with high precision. Collider experiments for *e*^+^*e*^−^, p$\bar{\text{p}}$, ep, pp colliders have played the major role for the searches and measurements of SM related phenomena. At the same time, verifying the SM also means searching for new physics beyond the SM or looking for the limits of the SM. These experiments have also made important contributions to constrain new physics beyond the SM.

Luminosity and energy upgrades of the hadron collider would certainly play important roles in looking for direct evidence of the new physics beyond the SM.

In parallel, studies of the detailed nature of the Higgs boson and exploring the mechanism of EW symmetry breaking are the important next steps for the future high-energy physics experiments, as they are the least studied part in the SM. Just as LEP played very important roles between the discoveries from the hadron colliders, the future higher-energy *e*^+^*e*^−^ collider would be an essential tool to open a window towards the Planck scale.

## Figures and Tables

**Table 1.** Fermions in the standard model as of 1974

particle	1-st generation	2-nd generation	3-rd generation	charge (*e*)
up-type quark	u	c	t	2/3
down-type quark	d	s	b	−1/3
neutrino	ν_*e*_	ν_μ_	ν_τ_	0
charged lepton	e	μ	τ	−1

Particle charges are in the unit of the elementary charge (*e*). Particles in the higher generation are heavier than the ones in the lower generation, except that neutrinos are assumed to be massless. The particles in hatched area were not found until 1974.

**Table 2.** Bosons in the standard model as of 1974

particle		spin	charge (*e*)	mass (GeV/*c*^2^)
photon	γ	1	0	0
gluon	*g*	1	0	0
charged weak boson	*W*	1	±1	80.385
neutral weak boson	*Z*	1	0	91.188
Higgs boson	*H*	0	0	125.1

The particles in hatched area were not found until 1974.

**Table 3.** Fermions in the standard model as of 1988

particle	1-st generation	2-nd generation	3-rd generation	charge (*e*)
up-type quark	u	c	t	2/3
down-type quark	d	s	b	−1/3
neutrino	ν_*e*_	ν_μ_	ν_τ_	0
charged lepton	e	μ	τ	−1

The particles in hatched area were not found until 1988.

**Table 4.** Bosons in the standard model as of 1988

particle		spin	charge (*e*)	mass (GeV/*c*^2^)
photon	γ	1	0	0
gluon	*g*	1	0	0
charged weak boson	*W*	1	±1	80.385
neutral weak boson	*Z*	1	0	91.188
Higgs boson	*H*	0	0	125.1

The particle in hatched area was not found until 1988.
